# A Prospective Longitudinal Study of the Clinical Outcomes from Cryptococcal Meningitis following Treatment Induction with 800 mg Oral Fluconazole in Blantyre, Malawi

**DOI:** 10.1371/journal.pone.0067311

**Published:** 2013-06-28

**Authors:** Camilla Rothe, Derek J. Sloan, Patrick Goodson, Jean Chikafa, Mavuto Mukaka, Brigitte Denis, Tom Harrison, Joep J. van Oosterhout, Robert S. Heyderman, David G. Lalloo, Theresa Allain, Nicholas A. Feasey

**Affiliations:** 1 Department of Medicine, University of Malawi, College of Medicine, Blantyre, Malawi; 2 Institute of Global Health, University of Liverpool, Liverpool, United Kingdom; 3 Liverpool School of Tropical Medicine, Liverpool, United Kingdom; 4 Queen Elizabeth Central Hospital, Ministry of Health, Blantyre, Malawi; 5 Malawi Liverpool Wellcome Trust Clinical Research Programme, University of Malawi College of Medicine, Blantyre, Malawi; 6 Infection and Immunity Research Centre, St. George’s Medical School, University of London, London, United Kingdom; 7 Department of Gastroenterology, University of Liverpool, Liverpool, United Kingdom; Institute of Infectious Diseases and Molecular Medicine, South Africa

## Abstract

**Introduction:**

Cryptococcal meningitis is the most common neurological infection in HIV infected patients in Sub Saharan Africa, where gold standard treatment with intravenous amphotericin B and 5 flucytosine is often unavailable or difficult to administer. Fluconazole monotherapy is frequently recommended in national guidelines but is a fungistatic drug compromised by uncertainty over optimal dosing and a paucity of clinical end-point outcome data.

**Methods:**

From July 2010 until March 2011, HIV infected adults with a first episode of cryptococcal meningitis were recruited at Queen Elizabeth Central Hospital, Blantyre, Malawi. Patients were treated with oral fluconazole monotherapy 800 mg daily, as per national guidelines. ART was started at 4 weeks. Outcomes and factors associated with treatment failure were assessed 4, 10 and 52 weeks after fluconazole initiation.

**Results:**

Sixty patients were recruited. 26/60 (43%) died by 4 weeks. 35/60 (58.0%) and 43/56 (77%) died or failed treatment by 10 or 52 weeks respectively. Reduced consciousness (Glasgow Coma Score <14 of 15), moderate/severe neurological disability (modified Rankin Score >3 of 5) and confusion (Abbreviated Mental Test Score <8 of 10) were all common at baseline and associated with death or treatment failure. ART prior to recruitment was not associated with better outcomes.

**Conclusions:**

Mortality and treatment failure from cryptococcal meningitis following initiation of treatment with 800 mg oral fluconazole is unacceptably high. To improve outcomes, there is an urgent need for better therapeutic strategies and point-of-care diagnostics, allowing earlier diagnosis before development of neurological deficit.

## Introduction

Cryptococcal meningitis, caused by the encapsulated saprophytic yeast *Cryptococcus neoformans*, occurs most commonly in HIV infected patients with CD4 counts of 100 cells/µl or less [1], [2]. As a consequence of the HIV epidemic, it is the most common cause of meningitis amongst adults in Sub Saharan Africa (SSA) [3]–[5]. Of an estimated global incidence of 957,900 cases per year, 75% occur in SSA [6].

Unacceptable differences in mortality exist between developed and developing countries. Ten week case fatality rates have been estimated at 9% in Western Europe and North America, but as high as 70% in SSA [6]. There is no evidence that mortality in SSA has improved since the roll-out of antiretroviral therapy (ART) and uncertainty over optimal treatment regimens for resource limited settings is partially responsible for this. ”Gold-standard” therapy involves a two week induction phase with the fungicidal combination amphotericin B and flucytosine prior to consolidation and maintenance therapy with fluconazole [7]. However, the cost, complexities of administration and side effects prevent the routine use of this regimen in resource poor settings. Standard treatment in Africa usually consists of fluconazole monotherapy.

Fluconazole is a fungistatic drug and past studies have shown poor clinical outcomes from monotherapy; in Uganda and Zambia administration of 200 mg/day resulted in 1–2 month survival of only 10–12% [8], [9]and in South Africa doubling the dose to 400 mg did not improve in-hospital survival [10]. A recent study demonstrated that dose escalation to 1200 mg/day achieves faster fungal clearance from cerebrospinal fluid (CSF) without increased toxicity [11], [12], but clinical end-point studies are lacking. At the time of the study, Malawian national guidelines stipulated the use of oral fluconazole at a dose of 800 mg [13].

Increasingly, early ART introduction has been advocated in patients suffering from opportunistic infections. Therefore, several African countries including Malawi now recommend commencing ART within a month of diagnosis of cryptococcal meningitis [13], [14]. However, patients who initiate ART very early (i.e. 3 days after starting 800 mg oral fluconazole) are at risk of intracranial Immune Reconstitution Inflammatory Syndrome (IRIS), which is often severe and can be fatal [15]. It is possible that patients initiated on fungistatic therapy are at greater risk of IRIS as the pathogen persists for longer. Conclusive data are lacking on the optimal time for initiating ART in these patients.

Within the last 5 years evidence has emerged that regimens incorporating short courses of amphotericin B may be safely and effectively used in South Africa and Uganda [16]–[20]. Clinical trials are now proposed using different combinations of amphotericin B, flucytosine and fluconazole to establish the best approach to treatment in resource poor settings. While preparations for these trials are underway, accurate clinical outcome data from a fluconazole-monotherapy regimen are required to provide a meaningful comparison from a high incidence and resource-poor setting.

From July 2010 until March 2011, 60 consecutive patients with confirmed cryptococcal meningitis in Blantyre, Malawi, were recruited and treated according to national guidelines [13]. They were followed-up for one year in order to determine clinical outcomes from cryptococcal meningitis in a resource limited setting following treatment induction with 800 mg oral fluconazole as monotherapy.

## Methods

### Ethics Statement

The study was prospectively approved by the University of Malawi, College of Medicine Research Ethics Committee (COMREC protocol P.04/10/926). Cryptococcal meningitis may impair a patient’s cognitive function to the extent that assistance of a guardian was required for discussions with the study team and therefore verbal consent was requested to approach all patients on diagnosis of the condition. Informed written consent to enter the study was obtained from patients or their guardians if unconscious. The study itself was completely observational: all procedures performed and medications commenced by the authors were consistent with their roles as attending physicians within the hospital. National and local guidelines and protocols for the management of cryptococcal meningitis and HIV were strictly adhered to by the study team in the routine care of the patients.

### Setting

Queen Elizabeth Central Hospital (QECH) is a 1,500 bed hospital in Blantyre, Malawi. It serves a population of approximately 1 million in the Blantyre District and takes tertiary referrals from further afield. Inpatient prevalence of HIV has been estimated at 70% amongst adult patients [21]. Since 2000, the Malawi Liverpool Wellcome Trust Clinical Research Programme (MLW) has offered a routine, quality controlled, diagnostic microbiology service for samples of cerebrospinal fluid (CSF) taken from adults and children presenting with clinical features suggestive of meningitis.

### Recruitment and Clinical Assessment

Patients were eligible for recruitment if they received a first microbiological diagnosis of cryptococcal meningitis following either positive India-Ink microscopy or positive culture of CSF. Malawian national guidelines for the treatment of Cryptococcal meningitis were followed; treatment was initiated with oral fluconazole 800 mg once daily (od) for 2 weeks, followed by 400 mg od for 6 weeks, then secondary prophylaxis with 200 mg od indefinitely [13].

Baseline demographic and clinical data were recorded including presence of fever (body temperature >37.5°C) and cranial nerve palsies or focal neurological signs. Prior use of ART and CD4 count at baseline were noted. Three aspects of overall neurological function were assessed; consciousness by Glasgow Coma Score (GCS), physical disability by modified Rankin Score (mRS) and cognition by Abbreviated Mental Test Score (AMTS). mRS is a 6 point scale (0 = No symptoms, 1 = No significant disability, 2 = Minor disability,3 = Moderate disability, 4 = Moderate-severe disability, 5 = Severe disability/bed-ridden), which has previously been used in Malawi for stroke patients [22]. AMTS is the sum of correct responses to 10 standard questions [23], adapted for Malawi by changing “When did World War I begin?” to “When did Malawi attain independence?” and replacing “Name the current UK monarch” with “Name the current Malawian president”. AMTS has not been validated in Malawi and results may be subject to bias by cultural background and educational attainment [24], however there was no better method of assessing cognition available.

It was not possible to measure CSF pressure and LPs were only repeated if patients developed clinical features of raised intra-cranial pressure.

Patients were reviewed 4, 10 and 52 weeks after initiation of fluconazole, and encouraged to contact the study team at any time if clinical deterioration occurred. ART was commenced 4 weeks into fluconazole therapy. The study team ensured continuous access to fluconazole and ART.

Treatment outcomes were defined as survival or treatment failure (death or therapy changed to amphotericin B following clinical deterioration with persistently positive CSF cultures after one month of fluconazole). This pragmatic approach to refractory cases was necessitated by a limited supply of amphotericin B, a shortage of trained staff to administer the drug and a lack of routine diagnostic biochemistry to monitor side effects.

### Laboratory Methods

All CSF diagnostic testing was performed at MLW. Cell counts were performed using a FastRead (Immune Systems) disposable counting chamber. India-Ink microscopy was performed on all unclotted samples of adequate volume. All samples were cultured on sheep blood and chocolate agar for 48 hours under aerobic and microaerophilic conditions and sub-cultured onto sabouraud agar and urea slopes if they were India-Ink positive or culture yielded yeasts. Bacteria and yeasts were identified using standard methods [25]. Neither cryptococcal antigen testing nor antifungal susceptibility testing was routinely available. Mycobacterial culture of CSF was not performed. The MLW laboratory participates in internationally recognised quality control programmes including NEQAS (UK) and the South African NHLS scheme. Full blood count and CD4 count were performed at the main QECH laboratory.

### Statistics

Clinical and microbiological data was stored in a Microsoft Excel file and analysed using “R” version 2.12.1. Baseline characteristics and treatment outcomes were reported by simple descriptive statistics. A two-sample Wilcoxon test was used to assess the relationship between baseline CD4 count and prior ART. Kaplan-Meier plots were used to demonstrate survival on fluconazole by 52 weeks. Data from previous studies suggest that deaths within the first month of treatment are directly attributable to cryptococcal meningitis whilst later deaths are multi-factorial [26]. Therefore, analysis of variables associated with poor outcome was done using Cox proportional hazards regression modelling with an end-point of “time to death” during the first four weeks. Hazard Ratios (HRs) with 95% confidence intervals (CI) were presented. This analysis was repeated using alternative end-points of “time to death or treatment failure” during 10 and 52 weeks of follow-up. In all analyses, a p-value of <0.05 was considered statistically significant.

## Results

### Baseline Demographic and Clinical Features

Sixty patients with a first episode of cryptococcal meningitis were recruited. Thirty-three (55%) were male and the median age was 32 years (range: 15–62). All patients complained of headache at presentation, with a median duration of 14 days (range: 1–150). 21 (37%) were febrile.

Baseline GCS was <14 of 15 in 14/58 (24%) patients. mRS was >3 (moderate disability) in 24/60 (40%) patients. 13/44 (30%) patients who completed an AMTS on admission obtained a score of <8/10. Five (10%) patients had localising neurological signs (two with focal weakness, two with cranial nerve VI palsies and one with both of these abnormalities).

All patients were HIV-infected. Thirty-two had a CD4 count result available, the median result was 37cells/µl (range: 2–234 cells/µl). Thirteen (22%) patients were on ART at presentation. Their median CD4 count was 55 cells/µl (range: 2–234cells/µl). There was no association between CD4 count and prior ART (two-sample Wilcoxon test, p = 0.69).

### Baseline CSF Characteristics

All 60 patients had evidence of cryptococcal infection in their CSF; 57 (95%) were culture positive and the remaining 3 (5%) had positive microscopy but no culture result available. White cells were seen in the CSF of 45 (75%) patients. The median white cell count was 20 cells/µl (range: 0–520).

Forty-six patients had a documented CSF protein result and 46 a documented CSF glucose. Median CSF protein was 0.78 g/L (range: 0.23–4.04, normal 0.15–0.40 g/L) and median CSF glucose was 1.97 mmol/L (range: 0.06–3.65, normal 2.22–3.88 mmol/L). 39/46 (85%) patients had high CSF protein and 27/44 (61%) had low CSF glucose values.

### Treatment Outcomes

26/60 (43%) patients died in the first 4 weeks. A total of 33 (55%) died by 10 weeks, and a further 2 failed fluconazole monotherapy so were switched to intravenous amphotericin B between weeks 2 and 10. In total, 35/60 (58%) patients had fluconazole treatment failure by 10 weeks.

**Figure 1:**
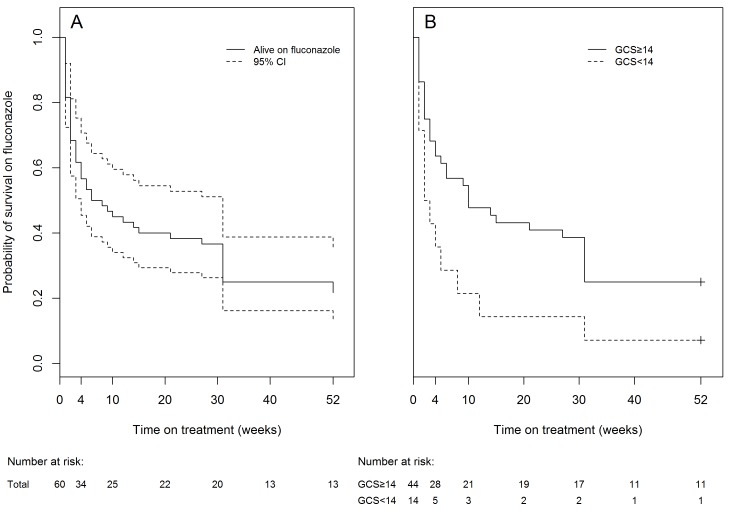
A shows survival on fluconazole therapy for all 60 patients. The solid line shows estimated proportion of survivors and the dotted lines show 95% confidence intervals. Amongst the 47 patients who did not survive on fluconazole to 52 weeks, two had positive CSF cultures at 10 weeks and were switched to fluconazole. The remaining 45 died. **B** shows survival on fluconazole stratified by baseline GCS. 2 patients (one who died in the first week and one who survived to 52 weeks) were excluded from this analysis as no baseline GCS was recorded. The solid line represents patients with baseline GCS≥14 and the dotted line represents patients with baseline GCS<14. Hazard ratio for death or treatment failure by 52 weeks: 2.09, 95% CI: 1.07–4.06 p = 0.030.

Between weeks 10 and 52, a further 8 patients died and 4 were lost to follow-up. There were only 13 known survivors one year after presentation. Of these, six were reviewed in person and seven were interviewed by telephone. The six presenting for review were taking both ART and secondary fluconazole prophylaxis. All had good neurological recovery (6 had AMTS = 10, 5 had mRS = 0, and one had mRS = 1). In total 43/56 (77%) of patients had failure of treatment of cryptococcal meningitis at one year following treatment induction with 800 mg oral fluconazole. Figure 1a shows that the probability of survival on fluconazole monotherapy for 4 weeks was 55% (95% CI: 44–70%), for 10 weeks was 43% (95% CI: 32–58%) and for one year was 22% (95% CI: 14–36%).

### Baseline Clinical Factors Associated with Treatment Outcome

Table 1 shows univariate analysis of associations between baseline variables and time to death during the first 4 weeks. Of the clinical factors, mRS>3 (HR: 3.00, 95% CI: 1.36–6.63) and AMTS score <8/10 (HR: 3.97, 95% CI: 1.37–11.50) were associated with shorter survival and there were strong trends towards earlier death with fever (HR: 2.27, 95% CI: 1.00–5.15) and GCS<14 (HR: 2.27 95%CI: 0.99–5.11). Of the laboratory factors, CSF glucose <2.2 mmol/l (HR: 3.13, 95% CI: 1.03–9.53) was associated with earlier death. Multivariate analysis was precluded by a high degree of co-linearity between clinical variables describing overall neurological function (mRS, AMTS and GCS), and the number of missing data-points amongst some laboratory variables.

**Table 1:** Baseline factors associated with treatment outcome by 4 weeks.

Variable	Total n = 60	Failed Treatment 1 n = 26	Survived n = 34	Hazard ratio (for death)		
				Hazard ratio	95% CI	p-value
***Demographics and clinical presentation***						
Age, years (median, IQR)	32 (29–39)	35 (28–40)	31 (29–37)	1.03	0.98–1.07	0.286
Male sex (n, %)	33 (55)	14 (54)	19 (56)	0.91	0.42–1.96	0.802
Headache duration, days (median, IQR)	14 (5–30)	14 (4–30)	17.5 (7–30)	1.00	0.99–1.02	0.707
Fever - temperature >37.5°C (n, %)	21 (37)	12 (52)	9 (27)	2.27	1.00–5.15	0.050
Cranial nerve palsy or localising neurological signs (n, %)	5 (10)	2 (8)	3 (9)	1.06	0.25–4.48	0.938
***Overall neurological function***						
Baseline GCS <14 (n, %)^2^	14 (24)	9 (36)	5 (15)	2.27	0.99–5.11	0.051
Modified Rankin Score (mRS) >3/5 (n, %)	24 (40)	16 (62)	8 (24)	3.00	1.36–6.63	0.007
Abbreviated Mental Test Score (AMTS) <8/10 (n,%)^3^	13 (30)	8 (57)	5 (17)	3.97	1.37–11.50	0.010
***HIV Parameters***						
On ART at baseline (n, %)^4^	13 (36)	6 (38)	7 (35)	1.21	0.44–3.34	0.709
***CSF parameters***						
≥5 White cells/mm^3^in CSF (n, %)	44(76)	20 (77)	25 (76)	1.18	0.46–0.72	0.724
White cell count in CSF (median cells/mm^3^, IQR)	20 (5–74)	18 (10–73)	29 (4–72)	1.00	1.00–1.00	0.787
CSF glucose below <2.2 mmol/l (n, %)^5^	27 (61.4)	14 (78)	13 (50)	3.13	1.03–9.53	0.045
CSF protein >0.4 g/L (n, %)^6^	39 (84.8)	16 (80)	23 (89)	0.71	0.24–2.14	0.546
CSF India Ink stain positive (n, %)	36 (61)	16 (61)	20 (61)	1.02	0.46–2.24	0.966

1 All treatment failures in the first 4 weeks died.

2 Baseline GCS known for 58 patients (25 failed, 33 survived).

3 Baseline AMTS performed on 44 patients (14 failed, 30 survived).

4 ART status known for 36 patients (n = 16 failed, n = 20 survived).

5 CSF glucose result available from 44 patients (18 failed, 26 survived).

6 CSF protein results available from 46 patients (20 failed, 26 survived).

When the univariate analysis was repeated with end-points of treatment failure by 10 or 52 weeks (Table 2), the importance of low CSF glucose concentration and fever at baseline were lost. However, on 10 week analysis AMTS<8/10 (HR: 2.96, 95%CI: 1.28–6.85), mRS>3 (HR: 2.96, 95%CI: 1.17–4.47) and GCS<14 (HR: 2.23, 95% CI: 1.08–4.62) were significantly associated with shorter survival. On 52 week analysis AMTS<8/10 (HR: 2.41, 95% CI: 1.07–4.06) and GCS<14 (HR: 2.09, 95% CI: 1.07–4.06) remained significant. Figure 1b demonstrates the probability of survival on fluconazole over 52 weeks sub-divided by baseline GCS<14 or >14.

**Table 2:** Baseline factors associated with treatment outcome by 10 and 52 weeks.

Variable	Treatment outcomes by 10 weeksHazard ratio (for death or failure)			Treatment outcomes by 52 weeksHazard ratio (for death or failure)		
	Hazard ratio	95% CI	p-value	Hazard ratio	95% CI	p-value
***Demographics and clinical presentation***						
Age, years (median, IQR)	1.01	0.96–1.05	0.758	1.01	0.96–1.05	0.803
Male sex (n, %)	0.69	0.35–1.34	0.273	0.70	0.39–1.29	0.254
Headache duration, days (median, IQR)	1.00	0.99–1.01	0.790	1.00	0.99–1.01	0.528
Fever - temperature >37.5°C (n, %)	1.47	0.72–2.98	0.295	1.27	0.66–2.41	0.469
Cranial nerve palsy or localising neurological signs (n, %)	0.76	0.18–3.18	0.709	1.78	0.62–4.94	0.285
***Overall neurological function***						
Baseline GCS <14	2.23	1.08–4.62	0.030	2.09	1.07–4.06	0.030
Modified Rankin Score (mRS) >3/5>3/5 (n, %)	2.28	1.17–4.47	0.011	1.76	0.96–3.22	0.068
Abbreviated Mental Test Score (AMTS) <8/10	2.96	1.28–6.85	0.011	2.41	1.12–5.19	0.024
***HIV Parameters***						
On ART at baseline (n, %)	1.46	0.62–3.42	0.385	1.47	0.68–3.19	0.326
***CSF parameters***						
>5 White cells/ml in CSF (n, %)	1.05	0.35–0.89-	0.984	0.93	0.49–1.77-	0.831
White cell count in CSF (cells/mm^3^)	1.00	0.99–1.01	0.841	0.99	0.99–1.00	0.676
CSF glucose below <2.2 mmol/l (n, %)^6^	1.81	0.77–4.25	0.172	1.29	0.63–2.62	0.483
CSF protein >0.5 g/L (n, %)	0.66	0.25–1.78	0.414	0.60	0.26–1.40	0.239
CSF India Ink stain positive (n, %)	0.82	0.42–1.60	0.559	0.86	0.47–1.56	0.607

Prior ART was not associated with better outcomes. Of the 13 patients on ART at baseline, 9 (69%) had died at 10 weeks and 11 (84%) at one year. As HIV viral loads, quantitative CSF fungal culture and CSF mycobacterial culture were unavailable, it was impossible to distinguish cryptococcal IRIS, HIV treatment failure and other causes of cerebral infection or IRIS.

CD4 counts were unavailable for 28 patients, 11 of who died within the first 4 weeks so were not used in the analysis.

## Discussion

Clinical outcomes from cryptococcal meningitis in HIV infected Malawian adults following treatment induction with 800 mg oral fluconazole were poor, with 43% mortality at 4 weeks, 58% treatment failure at 10 weeks and 77% treatment failure at 1 year. These data are consistent with prior studies of fluconazole monotherapy at both lower [8]–[10] and identical [11] doses in Africa and show only a modest improvement from the pre-ART and pre-fluconazole era. The 58% treatment failure rate at 10 weeks is similar to the 60% (18/30 patients) seen in a smaller cohort from a fluconazole dose response study in Uganda [11]. Malawi has since increased the induction phase dose of fluconazole to 1200 mg, although clinical end-point data from randomised controlled studies using this regimen are unavailable.

The contribution of baseline clinical and laboratory variables to poor outcomes by 4, 10 and 52 weeks was assessed. Although AMTS provides an imperfect assessment of cognitive function, a score of <8/10 was associated with earlier death or treatment failure at all time-points. Prior studies have reported similar results [26], suggesting that altered mental status at presentation is a persistently strong prognostic marker. Moderate or severe functional disability (mRS>3) and reduced consciousness (GCS<14), another marker of altered mental status were also associated with shorter survival by early and late time-points of analysis.

Fever (temperature >37.5°C) and low CSF glucose measurements (<2.2 mmol/l) were associated with poor outcomes by 4 weeks but were insignificant by later time-points. This finding is consistent with data suggesting the longer an individual survives, the less likely that subsequent death is due to cryptococcal meningitis [26]. Conversely, patients with impaired cognition, functional disability or reduced consciousness are at prolonged higher risk of poor adherence to medications and late treatment complications such as pressure sores, intercurrent infection and thrombosis. This may explain why baseline neurological deficit remained strongly linked to poor outcome after one year of follow-up.

The high incidence of altered mental status at baseline suggests that many patients presented with advanced disease. As the study contained no comparator arm, it is impossible to say whether better anti-fungal therapy would have improved outcomes in late presenters with worse neurological deficits. However, it is likely that public health interventions to encourage earlier presentation of patients with symptoms of meningitis will augment optimal anti-fungal treatments.

CD4 count data were incomplete, however several patients with extremely low CD4 counts survived to one year (median CD4 count amongst 13 long-term survivors: 13 (IQR 4–41) cells/µl). This illustrates the potential for full recovery from cryptococcal meningitis provided an adequate framework is in place to ensure uninterrupted supply of antifungals and timely commencement of ART. Without the intensive follow-up afforded by study participation, mortality in this group may have been higher.

Prior ART was not associated with improved survival and CD4 counts amongst ART-experienced patients were no higher than the rest of the cohort. This may represent initiation of ART during severe immune-suppression (i.e. CD4 count has risen from an unknown nadir) or ART failure. Irrespective, these data reinforce the well documented association between late ART initiation and higher mortality [27], [28] and emphasise the need for early HIV diagnosis and point of care cryptococcal antigen screening to detect early cryptococcal disease in high risk patients.

There were some limitations to this work. The sample size was small. As the study was conducted amidst routine clinical practice some data were incomplete, precluding multivariate analysis. Given the constraints of our setting, standardised management of high CSF pressure was not possible and it was difficult to characterise all co-morbidities, episodes of IRIS or causes of death after 4 weeks.

### Conclusions

We have described clinical outcomes from cryptococcal meningitis in patients initiated on 800 mg oral fluconazole monotherapy. Despite timely ART initiation and uninterrupted drug supply through near complete follow up, there was unacceptably high mortality. There is an urgent need to assess the efficacy of faster acting, combination fungicidal regimens, particularly in light of recent work on those containing flucytosine [29], [30]. In order to address this, a phase III trial assessing short-course amphotericin B and oral combination regimens will commence in 2013 [“Advancing cryptococcal meningitis treatment in Africa” (ACTA) ISRCTN: 45035509]. Improvements in clinical outcomes from cryptococcal meningitis must also involve public health interventions to encourage early presentation and roll-out of rapid, point of care diagnostics [31]. Earlier HIV diagnosis and ART initiation is of equal importance to prevent all diseases of advanced immunosuppression [32].
